# Does Transglutaminase Crosslinking Reduce the Antibody Recognition Capacity of β-Lactoglobulin: An Analysis from Conformational Perspective

**DOI:** 10.3390/molecules30030685

**Published:** 2025-02-04

**Authors:** Lei Fang, Xun Han, Yue Zhang, Tianran Hui, Lingling Ding, Wenlu Dai, Yujie Han, Maoqiang Zheng, Guangliang Xing

**Affiliations:** 1 School of Biology and Food Engineering, Changshu Institute of Technology, Changshu 215500, China; 2 UCL Division of Medicine, University College London, London WC1E 6BT, UK

**Keywords:** β-lactoglobulin, transglutaminase, antibody recognition, conformational changes

## Abstract

Food allergies are a global concern, with β-lactoglobulin (β-LG) in bovine milk being a major allergenic protein. This study investigated the effects of transglutaminase (TGase)-mediated crosslinking on the antibody recognition capacity (ARC) and structural properties of β-LG, with the aim of developing hypoallergenic dairy products. β-LG solutions were treated with TGase at varying concentrations (0, 5, 10, 15, and 20 U/g) and durations (0, 6, 18, 24, and 42 h), followed by analysis using electrophoresis, enzyme-linked immunosorbent assay (ELISA), and spectroscopy. The results demonstrated that treatment with TGase at 20 U/g significantly reduced the ARC and immunoglobulin E (IgE) binding capacity of β-LG to 90.0 ± 0.4% and 58.4 ± 1.0%, respectively, with the optimal ARC reduction observed after 6 h of treatment (86.7 ± 1.2%, *p* < 0.05). Although electrophoresis did not reveal significant crosslinking of β-LG, ultraviolet absorption, fluorescence intensity, and hydrophobicity all increased with prolonged crosslinking time, while sulfhydryl content fluctuated irregularly. These findings suggest that β-LG underwent varying degrees of structural modification, which led to the masking of antigenic epitopes during the early stages (0–24 h), followed by their re-exposure at the later stage (42 h). Overall, these results highlight the potential of TGase to reduce β-LG potential allergenicity, presenting a promising strategy for the development of hypoallergenic dairy products.

## 1. Introduction

Whey protein, a component of bovine milk renowned for its nutritional benefits and functional properties, is a vital ingredient in the food processing industry. Its superior emulsifying, gelling, and foaming abilities, along with its high nutritional value, allow for its incorporation into a variety of food products, including infant formulas, protein supplements, and dairy-based functional foods [1]. β-LG is the major whey protein, accounting for approximately 50% of the total whey protein content. This is significant because β-LG is not present in human milk, which categorizes it as an exogenous protein [2]. As a result, β-LG is more prone to inducing allergic reactions in sensitized individuals compared to other proteins. It has been reported that approximately 82% of individuals with milk allergies experience severe allergic reactions to β-LG [3]. Allergic responses to β-LG can range from mild symptoms, such as skin rashes and gastrointestinal discomfort, to severe reactions, such as anaphylactic shock, which can be life-threatening [4]. Therefore, reducing its allergenicity has emerged as a critical area of research in recent years.

Several strategies have been explored to mitigate the allergenicity of β-LG. Conventional methods, including heat treatment, enzymatic hydrolysis, and fermentation, are commonly employed to modify its structure and reduce immunogenicity. Bu et al. [5] investigated the heat-induced antigenicity changes of β-LG in milk and found that heating to 90 °C caused β-LG molecules to unfold, exposing conformational epitopes and increasing their sensitivity to hydrolysis, which, in turn, heightened the allergic response. However, further heating to 100 °C and 120 °C masked or destroyed these epitopes, thereby reducing allergenicity. Villas-Boas et al. [6] demonstrated that hydrolysis of β-LG by either alcalase or bromelain effectively cleaved its antigenic epitopes, thereby reducing its IgE binding capacity. Pescuma et al. [7] found that proteases produced during the fermentation of lactic acid bacteria could degrade β-LG epitopes, thereby reducing allergic responses. Physical treatments, including high-pressure processing and irradiation, have also been explored for modifying β-LG epitopes. Bogahawaththa et al. [8] investigated the impact of high-pressure processing on skim milk and found that high-pressure-induced protein aggregation through thiol-disulfide interactions led to structural changes and protein denaturation, which decreased immunogenicity. Yang et al. [9] observed that irradiation of β-LG samples with a Co-60 irradiator at a dose of 5 kGy increased aggregation, reduced surface hydrophobicity, and inhibited β-LG-induced Ca^2^⁺ influx, thereby attenuating IgE-mediated allergic reactions. However, these methods often compromise the functional properties of β-LG, such as its emulsifying and foaming abilities, highlighting the need for more innovative and efficient strategies.

TGase, an enzyme widely utilized in the food industry, presents a promising alternative for reducing β-LG allergenicity. By catalyzing covalent crosslinking between protein molecules, TGase induces structural modifications that mask allergenic epitopes while preserving the functional characteristics of β-LG. This enzymatic approach has been successfully applied to reduce the immunoreactivity of allergenic proteins, including soy protein, ovomucoid, *Penaeus chinensis* tropomyosin, and milk protein [10,11,12,13,14], demonstrating its potential for mitigating β-LG allergenicity. In this study, we examined the effects of TGase-mediated crosslinking on the ARC and structural properties of β-LG. By elucidating the relationship between enzymatic modification and ARC, this research aims to contribute to the development of safe, hypoallergenic dairy products.

## 2. Results and Discussion

### 2.1. Changes in Electrophoretic Profile, ARC, and IgE Binding Capacity of β-LG Crosslinked by Varying TGase Concentrations

**Figure 1:**
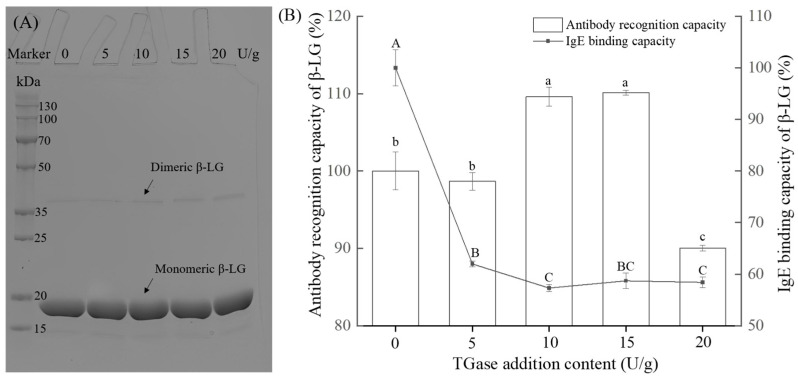
Effect of TGase-mediated crosslinking at varying concentrations (0 (control), 5, 10, 15, and 20 U/g) on the electrophoretic profile (**A**), ARC and IgE binding capacity (**B**) of β-LG. Different lowercase letters (a–c) indicate a significant difference (*p* < 0.05) in ARC, and different uppercase letters (A–C) letters indicate a significant difference (*p* < 0.05) in IgE binding capacity.

Figure 1 illustrates the effects of TGase-mediated crosslinking at varying concentrations (0, 5, 10, 15, and 20 U/g) on the electrophoretic profile, ARC, and IgE binding capacity of β-LG. The sodium dodecyl sulfate-polyacrylamide gel electrophoresis (SDS-PAGE) results (Figure 1A) show that the intensity of the monomeric β-LG band (~18 kDa) and dimeric β-LG band (~38 kDa) remained largely unchanged as the TGase concentration increased. This is attributed to the compact, globular structure of β-LG, which limits the efficiency of crosslinking [15]. Benedé et al. [16] observed similar results, noting that β-LG monomers persisted in the electrophoretic gel even after extensive TGase treatment at a concentration of 1000 U/g of protein. ARC analysis (Figure 1B) reveals a non-linear response to TGase treatment. At lower TGase concentrations (5 U/g), ARC remained largely unchanged compared to the control (*p* > 0.05). At moderate concentrations (10–15 U/g), ARC significantly increased (*p* < 0.05), reaching 109.6 ± 1.2% and 110.1 ± 0.3%. This enhancement may result from TGase-induced conformational changes that expose previously hidden epitopes, thereby increasing antibody binding affinity [17]. Villas-Boas et al. [18] also noted that heat-treated β-LG subjected to TGase polymerization exhibited increased antigenicity compared to its native form. However, at the highest TGase concentration (20 U/g), ARC decreased significantly to 90.0 ± 0.4% (*p* < 0.05), suggesting that further increases in TGase concentration obscured critical epitopes, reducing their accessibility to antibodies. The IgE binding capacity of β-LG significantly decreased after crosslinking with varying amounts of TGase, with the lowest IgE binding capacity observed at a TGase concentration of 10 U/g (57.3 ± 0.7%). No significant difference in IgE binding capacity was observed between the 10 and 20 U/g TGase (58.4 ± 1.0%) concentrations (*p* > 0.05). These results indicate that TGase concentration modulated the structural and antigenic properties of β-LG. The interaction between TGase concentration and the structural modifications of β-LG highlights the need for a balance to optimize ARC [17]. Based on the ARC and IgE binding capacity results, we applied 20 U/g of TGase to crosslink β-LG and further investigated the effect of crosslinking time on its ARC and conformation.

### 2.2. SDS-PAGE Profiles and ARC of β-LG Crosslinked by TGase over Time

**Figure 2:**
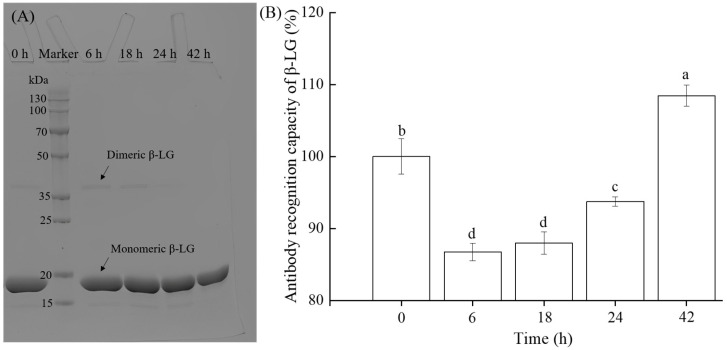
Effect of TGase-mediated crosslinking at varying durations (0 (control), 6, 18, 24, and 42 h) on the electrophoretic profile (**A**) and ARC (**B**) of β-LG. Different lowercase letters indicate a significant difference (*p* < 0.05) in ARC.

The findings depicted in Figure 2 reveal the time-dependent effects of TGase treatment on the crosslinking and ARC of β-LG. In Figure 2A, SDS-PAGE analysis shows that the monomeric β-LG (~18 kDa) remains present even after crosslinking with TGase (20 U/g) for 42 h, with no significant change in density. However, at 42 h, the dimeric β-LG band (~38 kDa) disappeared due to crosslinking by TGase [19]. Figure 2B illustrates the time-dependent changes in the ARC of β-LG treated with TGase. Initially, ARC decreased significantly (*p* < 0.05) at 6 h (86.7 ± 1.2%) and remained low at 18 h (88.0 ± 1.6%), indicating that early-stage crosslinking masked critical antibody-binding epitopes. However, as the reaction progressed to 24 h, ARC slightly recovered (93.7 ± 0.6%), suggesting structural rearrangements or partial aggregate formation that exposed limited epitopes. By 42 h, ARC increased dramatically to 108.5 ± 1.5% (*p* < 0.05), surpassing the control level. This increase was likely attributed to the formation of large protein aggregates or extensive structural modifications that exposed previously hidden epitopes, making them more accessible to antibodies [20]. The observed biphasic trend in ARC aligns with previous studies showing that TGase-mediated crosslinking can alter protein conformation and epitope accessibility [13,21]. Early-stage crosslinking may stabilize the protein structure, reducing epitope availability, while prolonged treatment could induce substantial modifications that unmask new epitopes or alter protein structure to enhance immunogenicity. Such findings have important implications for food processing, particularly concerning the allergenicity of milk proteins. Furthermore, as shown in Figure 1 and Figure 2, changes in the density of β-LG bands do not necessarily correlate with its ARC, as conformational changes may mask allergenic epitopes, thus evading detection by SDS-PAGE [22,23]. Further investigation into structural alterations during TGase-mediated crosslinking could enhance our understanding of ARC modifications.

### 2.3. Fourier Transform Infrared (FTIR) Spectroscopy Analysis

**Figure 3:**
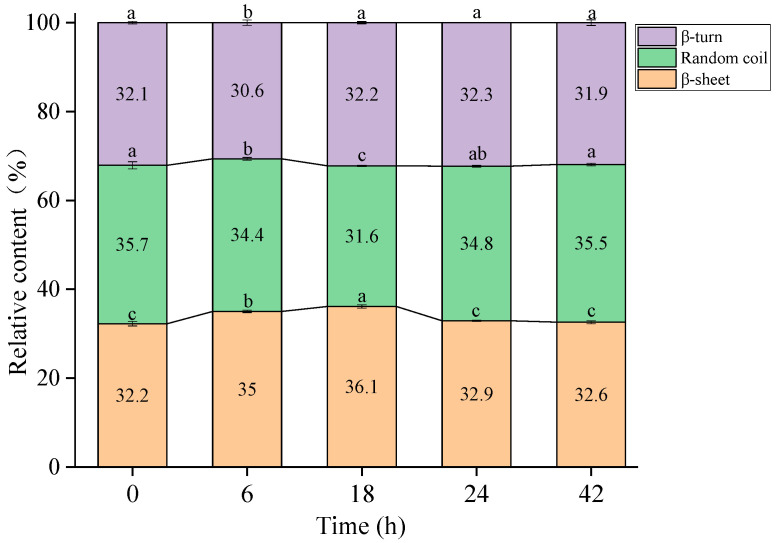
The relative proportions of protein secondary structures in β-LG samples following TGase crosslinking with varying durations (0 (control), 6, 18, 24, and 42 h). Different lowercase letters (a–c) indicate a significant difference (*p* < 0.05).

The characteristic absorption bands indicative of protein secondary structure are predominantly observed in the amide I region, spanning from 1700 to 1600 cm^−1^. These bands are primarily attributable to the C=O stretching vibrations, with contributions from the in-plane N-H bending and C-H stretching modes, which reflect changes in the secondary structure composition of proteins, including α-helix, β-sheet, β-turn, and random coil configurations [24]. To comprehensively understand the conformational and aggregation characteristics of β-LG, alterations in its secondary structure before and after exposure to TGase over various durations were examined. The secondary structure of β-LG exhibited dynamic alterations during TGase-mediated crosslinking, as shown in Figure 3. Specifically, there was a significant increase in β-sheet content from 32.2 ± 0.5% at 0 h to 36.1 ± 0.4% at 18 h (*p* < 0.05), indicating enhanced structural stabilization due to the covalent bonding between lysine and glutamine residues facilitated by TGase. These covalent bonds promoted β-sheet formation, contributing to protein aggregation, consistent with previous findings on TGase-catalyzed crosslinking of whey protein isolate [25]. A similar result was reported that the crosslinking activity of TGase significantly enhanced the β-sheet content of surimi proteins that had been subjected to high-pressure processing prior to treatment [26]. However, prolonged incubation (24–48 h) resulted in a decrease in β-sheet content to 32.6 ± 0.3%, accompanied by an increase in random coil structures to 35.5 ± 0.3%, indicating a transition towards more disordered conformations [27]. This reduction may be attributed to the formation of extensive protein aggregates, which disrupt the ordered β-sheet networks. Interestingly, β-turn content remained relatively stable (~32%), implying that β-turn structures were less susceptible to the conformational changes induced by TGase crosslinking. The biphasic behavior observed here highlights that crosslinking initially stabilized β-LG’s secondary structure but, at longer reaction times, may induce structural disruption due to extensive aggregation. These findings offer valuable insights into the structural consequences of TGase crosslinking on β-LG, with potential implications for its functional properties in food systems.

### 2.4. Ultraviolet (UV) Absorption Spectra

**Figure 4:**
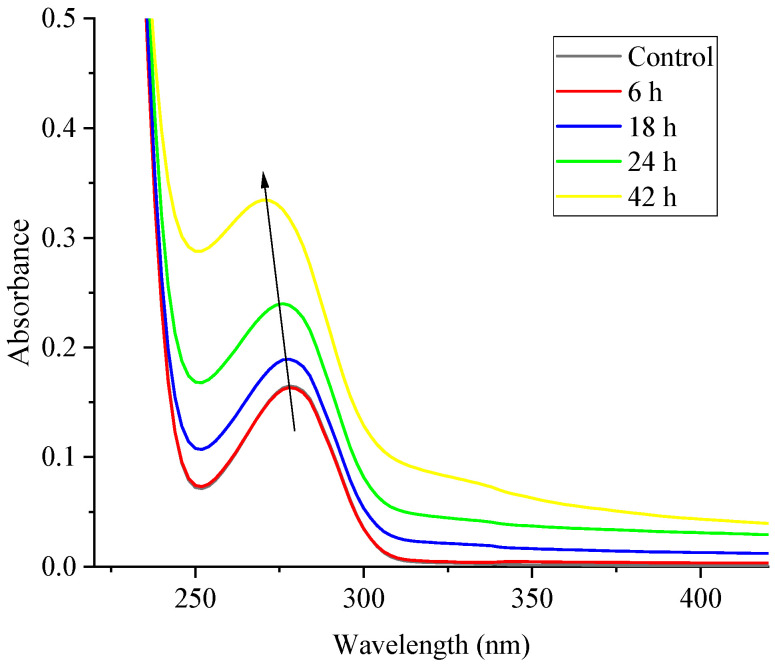
The ultraviolet absorption spectra of the β-LG samples following TGase crosslinking with varying durations (0 (control), 6, 18, 24, and 42 h). The arrow indicates a blue shift in the λ_max_.

Some aromatic amino acid residues in β-LG, including tryptophan, tyrosine, and phenylalanine, have a maximum absorption peak in the UV region at around 280 nm [28]. Changes in the tertiary structure of β-LG can be inferred from shifts in the UV absorption spectra of samples that underwent crosslinking with TGase over different time periods. These spectra provide valuable insights into the structural changes occurring during TGase-mediated crosslinking, as illustrated in Figure 4. At 6 h, the UV absorption intensity of β-LG remained largely unchanged compared with the control (0 h); however, as the crosslinking time increased, the absorption intensity gradually increased. By 18 and 24 h, the absorbance increased progressively, suggesting partial structural rearrangements or localized unfolding that exposed previously buried aromatic residues. At 42 h, a sharp increase in UV absorbance was observed, significantly surpassing that of the control (0 h), indicating extensive structural disruptions and aggregation caused by prolonged TGase treatment, which exposed aromatic residues to the solvent. Additionally, a slight blue shift in the maximum UV absorbance (λ_max_) was observed, from 278 nm at 0 h, 6 h, and 18 h to 276 nm at 24 h and 270 nm at 48 h. Generally, a blue shift occurs when aromatic residues, particularly tryptophan, are exposed on the protein surface, resulting in changes to the polarity of their surrounding microenvironment [29]. The changes in the tertiary structure observed here are consistent with the findings of Zhu et al. [30] who reported similar results in UV absorption spectra of TGase-crosslinked soy protein, noting the exposure of aromatic amino acids and an increase in UV absorbance. These findings align with previous research indicating that enzymatic crosslinking can alter the tertiary structure of proteins, leading to the partial unfolding of internal peptide chains and a more extended conformation, which, in turn, exposes more tryptophan and tyrosine residues to UV light [31]. The increased absorbance at longer crosslinking times correlates with the formation of large protein aggregates, as observed in SDS-PAGE analyses. Understanding the conformational changes in β-LG during TGase crosslinking is crucial for optimizing its functional properties in food processing while minimizing potential allergenicity, such as excessive aggregation or altered allergenicity.

### 2.5. Fluorescence Spectroscopy

**Figure 5:**
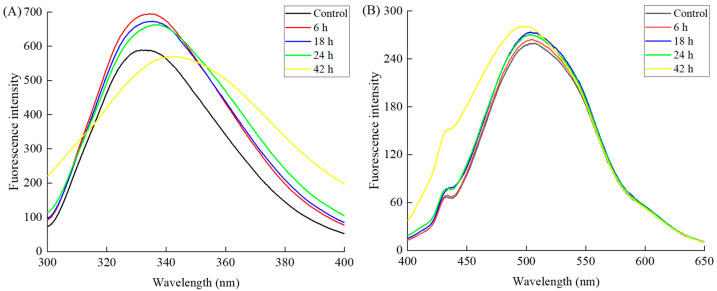
The intrinsic fluorescence spectra (**A**) and extrinsic fluorescence spectra (**B**) of the β-LG samples following TGase crosslinking with varying durations (0 (control), 6, 18, 24, and 42 h).

Fluorescence intensity serves as a vital indicator of protein tertiary structure, reflecting the environment around aromatic amino acids, particularly tryptophan residues, which are sensitive to changes in protein conformation [32]. The intrinsic fluorescence spectra of β-LG after TGase crosslinking revealed significant time-dependent structural changes, as shown in Figure 5A. At 6 h, the fluorescence intensity of β-LG increased compared to the control (0 h), indicating enhanced solvent exposure of tryptophan residues due to moderate conformational changes. This trend continued at 18 and 24 h, with fluorescence remaining elevated relative to the control, suggesting sustained destabilization of the native β-LG structure. These results suggest that short-term TGase crosslinking (0–24 h) may expose previously buried tryptophan residues in β-LG, consistent with prior research [33], which reported a slight increase in fluorescence intensity of TGase-treated whey protein isolate compared to untreated samples. However, at 42 h, fluorescence intensity sharply decreased, accompanied by a red shift in the emission peak (from 331.7 nm at 0 h to 342.4 nm at 48 h). This reduction indicates substantial structural alterations, likely due to protein unfolding, which exposes tryptophan residues to a more polar environment. Yang and Xiong [34] also reported a decrease in the fluorescence intensity of tryptophan residues in a polar environment due to protein unfolding. These findings align with a previous study suggesting that TGase crosslinking initially stabilized protein structures through covalent bond formation but, over time, promoted structural destabilization [35]. The observed changes in β-LG fluorescence have practical implications for food systems, where TGase-induced modifications are used to reduce allergenicity. Optimizing the crosslinking duration is crucial for achieving the desired protein properties while minimizing aggregation levels that could lead to altered protein conformation or functional loss.

The extrinsic fluorescence spectra (Figure 5B) reflect changes in the hydrophobic surface exposure of β-LG during TGase-mediated crosslinking. β-LG contains a significant number of hydrophobic amino acid residues located internally [36]. Initially, the native β-LG structure (control) exhibited low fluorescence intensity, consistent with limited exposure of hydrophobic regions in its compact conformation. At 6, 18, and 24 h, the fluorescence intensity increased slightly, suggesting early conformational changes induced by TGase crosslinking. During this period, TGase catalyzed the formation of covalent bonds between lysine and glutamine residues, stabilizing the β-LG structure and causing minor rearrangements that exposed limited hydrophobic regions [37]. After 42 h of TGase treatment, the fluorescence intensity increased to approximately 290, reflecting substantial exposure of hydrophobic regions. This significant increase indicates protein unfolding, where hydrophobic core regions become solvent-accessible. These findings are consistent with a previous study reporting increased surface hydrophobicity and protein aggregation in TGase-crosslinked whey protein-soluble aggregates [38].

### 2.6. Free Sulfhydryl (-SH) Group Content Analysis

**Figure 6:**
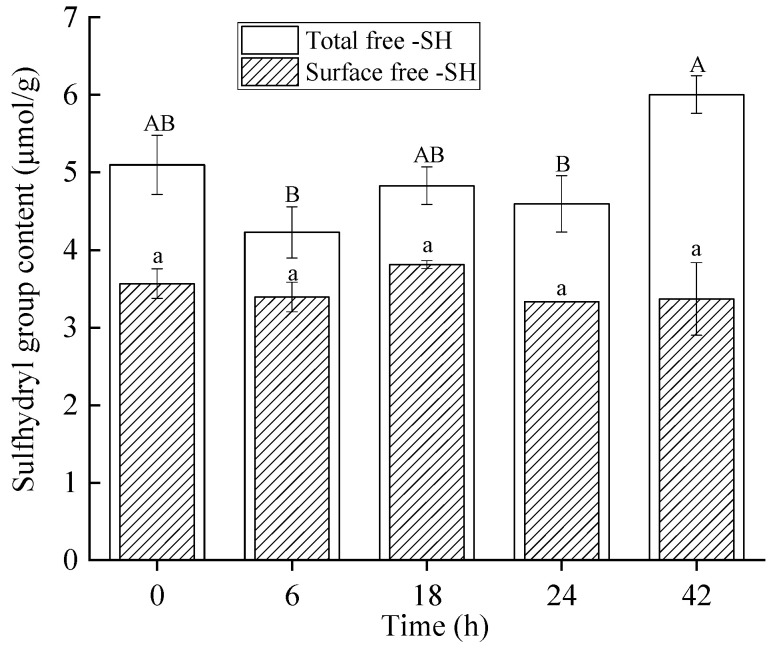
The total free SH content and surface free -SH of the β-LG samples following TGase crosslinking with varying durations (0 (control), 6, 18, 24, and 42 h). Same lowercase letters denote no significant differences in surface free -SH content (*p* > 0.05), while different capital letters (A, B) indicate significant differences in total free -SH content (*p* < 0.05).

Sulfhydryl groups are critical for proteins, as they facilitate the formation of disulfide bonds, thereby stabilizing higher-order structures and influencing functional properties [39]. The analysis of sulfhydryl groups in β-LG during TGase crosslinking revealed a dynamic balance between structural stabilization and disruption, as shown in Figure 6. Initially, the total free -SH content decreased from 5.1 μmol/g protein (control) to 4.2 μmol/g protein at 6 h, suggesting that TGase crosslinking stabilized the β-LG structure by promoting covalent interactions involving lysine and glutamine residues. This stabilization likely reduced the availability of sulfhydryl groups, either through structural masking or their involvement in intramolecular interactions [40]. Interestingly, at 48 h, the total free -SH content significantly increased to 6.0 μmol/g protein, indicating structural rearrangements or unfolding due to prolonged TGase treatment. These results were consistent with the findings from UV spectroscopy (Figure 4) and fluorescence spectroscopy (Figure 5). This unfolding could expose previously buried sulfhydryl groups, a phenomenon commonly observed during protein aggregation or denaturation [32,41]. However, the surface free -SH content remained relatively stable (3.4–3.8 μmol/g protein) throughout the experiment (*p* > 0.05), suggesting that the free sulfhydryl groups were not extensively consumed during TGase-mediated reactions. Instead, TGase primarily facilitated covalent crosslinking via lysine and glutamine residues, with limited involvement of sulfhydryl groups. Understanding how TGase modulates the availability of sulfhydryl groups provides valuable insights into its impact on β-LG structure and functionality, which is essential for optimizing protein modification processes in food applications, particularly in reducing allergenicity.

## 3. Materials and Methods

### 3.1. Materials

β-LG, with a purity of 95% (*w*/*w*), was procured from Aladdin Biochemical Technology Co., Ltd., Shanghai, China. TGase, characterized by an enzyme activity of 100 U/g, was acquired from Dongsheng Bio-tech Co., Ltd., Jiangsu, China. For the immunoassay, the β-LG-specific enzyme-linked immunosorbent assay (ELISA) kit was provided by Shanghai Enzyme-linked Biotechnology Co., Ltd., Shanghai, China. Unless explicitly stated otherwise, all additional reagents employed throughout the experimental procedures were of analytical grade.

### 3.2. Preparation of TGase-Crosslinked β-LG Samples

A specified amount of β-LG was weighed and fully dissolved in phosphate-buffered saline (PBS, pH 6.8, 0.05 mol/L) to achieve a protein concentration of 1.0 mg/mL. To examine the effect of TGase concentration on the ARC of β-LG, the solution was divided into five groups, with each receiving a different amount of TGase (0, 5, 10, 15, or 20 U/g protein). The samples underwent incubation at 50 °C for 24 h, followed by thermal inactivation of the enzyme at 85 °C for 5 min, and were then refrigerated at 4 °C. In order to investigate the influence of crosslinking duration on the ARC and conformation of β-LG, a new batch of β-LG solution (1.0 mg/mL) was prepared and divided into five groups, each supplemented with 20 U/g of TGase. These groups were incubated at 50 °C for 0, 6, 18, 24, or 42 h, followed by the same enzyme inactivation process and subsequent storage at 4 °C for further analysis.

### 3.3. SDS-PAGE

To evaluate the molecular weight variations of β-LG across different treatment groups, protein gel electrophoresis was performed using a 12% (*w*/*v*) separating gel and a 4% (*w*/*v*) stacking gel [42]. Each sample, 20 µL in volume, was combined with 5 µL of 5 × loading buffer (QS05028, provided by Jiangsu Qianzhusong Biological Technology Co., Ltd., Jiangsu, China) and subjected to heat at 95 °C for 5 min. Then, 10 µL of each sample was introduced into the gel wells. SDS-PAGE was conducted under constant voltage, initially at 60 V for 1 h, followed by an increase to 120 V. Once the electrophoresis was completed, the gel was stained and destained before being analyzed using Quantity One software version 4.6.2.

### 3.4. Indirect Competitive ELISA

The ARCof β-LG was assessed employing a bovine β-LG sandwich ELISA kit. Reagents were retrieved from 4 °C storage, and the β-LG concentrations in the different treatment groups were adjusted to 200 µg/L. The assay setup included blank wells (without sample or enzyme-conjugated reagent), standard wells, and sample wells. To the standard wells, 50 µL of standards at a range of concentrations were introduced. In the sample wells, 40 µL of sample dilution buffer was initially pipetted, followed by the addition of 10 µL of the sample (achieving a final dilution ratio of 5×). The plate was then sealed and incubated at 37 °C for a duration of 30 min. Afterward, 200 µL of washing buffer was dispensed into each well and incubated for 30 s, and this washing process was reiterated five times. Horseradish peroxidase-conjugated reagent (50 µL) was then added to each well, with further incubation for an additional 30 min. The wells were washed five more times with the washing buffer. Subsequently, 100 µL of chromogenic reagent was introduced, and the plate was incubated at 37 °C for 15 min in darkness. The reaction was stopped by the addition of 50 µL of stop solution, and the absorbance was recorded at 450 nm. The concentrations of β-LG were determined using a standard curve, and the effect of TGase crosslinking on the ARC of β-LG was assessed by comparing the levels of β-LG in crosslinked samples with those in untreated control samples. Measurements were performed in duplicate.

The IgE binding capacity of β-LG in non-crosslinked and varying concentrations of TGase-treated samples was evaluated using human serum from patients with cow milk allergies, as previously stated by Xing et al. [17]. The specific IgE levels in the sera of three milk-allergic patients (aged 24, 31, and 68 years) were 6.2, 12.4, and 75.1 kU/L, respectively. These sera were provided by PlasmaLab International (Everett, WA, USA). Informed consent was obtained from all participants, and they confirmed that their sera could be used for milk allergen research. IgE reactivity was determined as follows:

IgE binding capacity (%) = (OD_450_ of TGase-treated β-LG samples/OD_450_ of untreated β-LG samples) × 100%. (1)

### 3.5. FTIR Spectroscopy Analysis

The β-LG samples from different treatment groups were freeze-dried and then mixed with spectroscopic-grade KBr (previously dried at 130 °C for 30 min) at a mass ratio of 1:100. The mixture was ground in an agate mortar and pressed into thin, transparent sheets. FTIR spectra were recorded using a Fourier transform infrared spectrometer (FTIR-650, Gangdong Sci. & Tech. Co., Ltd., Tianjin, China). Each sheet was scanned a total of 32 times over a range of 4000 to 400 cm^−1^ at a resolution of 4 cm^−1^. The amide I region, spanning from 1700 to 1600 cm^−1^, was specifically selected for detailed examination [25]. The acquired data were subsequently processed using Peak Fit v4.12 and Omnic 8.0 software to assess alterations in the secondary structure of β-LG among the various treatment groups.

### 3.6. UV Absorption Spectroscopy

A 1 mL aliquot of β-LG from each treatment group was diluted fivefold with PBS (pH 6.8, 0.05 M), yielding a final concentration of 0.2 mg/mL. A 3 mL aliquot of each diluted solution was then placed into a quartz cuvette for UV spectral analysis, which was conducted using a UV-1800 PC spectrophotometer from Shanghai Meipuda Instrument Co., Ltd., Shanghai, China. The wavelength range for scanning was set from 220 to 420 nm [43].

### 3.7. Intrinsic Fluorescence Spectroscopy

The samples were diluted to achieve a β-LG concentration of 0.2 mg/mL using PBS (pH 6.8, 0.05 M). Intrinsic fluorescence intensity was measured using an F-280 fluorescence spectrophotometer supplied by Gangdong Sci. & Tech. Co., Ltd., Tianjin, China. For the measurements, the excitation wavelength was fixed at 280 nm, with the emission wavelength range set between 300 and 400 nm. The slit widths for both excitation and emission were standardized to 5 nm [44].

### 3.8. Surface Hydrophobicity Measurement

The surface hydrophobicity of the samples was assessed using the 1-anilinonaphthalene-8-sulfonate (ANS) fluorescence probe method, with modifications based on Luo et al. [37]. β-LG samples were diluted to a concentration of 0.2 mg/mL using PBS (pH 6.8, 0.05 M). To 4 mL of the diluted sample, 20 μL of ANS solution (8.0 mmol/L) was introduced, and the mixture was vigorously stirred and then incubated in the absence of light at ambient temperature for 20 min. Fluorescence intensity was recorded at an excitation wavelength of 390 nm and an emission wavelength range of 400 to 650 nm, with a scanning speed of 240 nm/s and a slit width of 5 nm. Hydrophobicity was expressed as fluorescence intensity.

### 3.9. Determination of Free -SH Groups

The concentration of free -SH groups in β-LG samples across different treatment groups was determined, adhering to the protocol established by Zhang et al. [38] with minor modifications. A 15 mg lyophilized sample was dissolved in 5 mL of Tris-glycine buffer, which contained 0.086 mol/L Tris, 0.09 mol/L glycine, and 4 mmol/L ethylenediaminetetraacetic acid (EDTA), for the quantification of surface -SH groups. For total free -SH group determination, the same buffer was used but with the addition of 8 mol/L urea. To each solution, 50 µL of Ellman’s reagent (prepared by dissolving 4 mg of 5,5′-dithiobis-(2-nitrobenzoic acid) in 1 mL of the corresponding Tris-glycine buffer) was introduced. After vortexing, the suspension was incubated in the dark at room temperature for 30 min. Absorbance at 412 nm (A_412_) was determined, with the blank containing only the buffer without protein. The -SH group concentration was calculated using the following formula:

-SH (µmol/g) = (73.53 × A_412_ × D)/C (2)

where -SH denotes the content of surface or total free sulfhydryl groups in the protein, A_412_ is the absorbance measured at 412 nm, D represents the dilution factor, and C is the concentration of β-LG in milligrams per milliliter.

### 3.10. Statistical Analysis

Each experimental trial was conducted three times to confirm the reliability of the findings, with the outcomes expressed as the mean ± standard deviation. The statistical evaluation was performed using a one-way analysis of variance (ANOVA), complemented by Duncan’s post hoc test for multiple comparisons, with the aid of SPSS software (version 16.0, Chicago, IL, USA). Statistical significance was defined as *p* < 0.05.

## 4. Conclusions

This study demonstrates the dual impact of TGase-mediated crosslinking on the ARC and structural properties of β-LG. Moderate enzyme concentrations (10–15 U/g) and prolonged treatment time (up to 42 h) enhanced ARC, which might be due to the exposure of hidden epitopes. However, short-term treatment with higher enzyme concentration (20 U/g for 6–18 h) resulted in significant structural disruption and reduced ARC, potentially masking allergenic epitopes. Secondary structure analysis revealed dynamic changes, including an initial increase in β-sheet content followed by a dominance of random coil structures, indicating protein destabilization and aggregation. UV and fluorescence analyses further confirmed the exposure of buried aromatic residues and sulfhydryl groups during extended treatment, suggesting structural unfolding. These findings underscore the critical importance of optimizing TGase concentration and reaction time to achieve desired structural and functional modifications while minimizing adverse effects on allergenicity. By leveraging TGase-mediated crosslinking, it is possible to develop hypoallergenic whey-based food products with preserved functional properties, offering significant potential for applications in the dairy and food industries. Further research into the precise mechanisms of TGase interaction with β-LG will enhance the understanding and improve the application of this enzymatic approach in reducing milk protein allergenicity.

## Data Availability

The data presented in this study are available on request from the corresponding author due to reasonable request.
